# Infrastructure, capabilities, and capacities required for clinical trials design and delivery: A rapid scoping review of recommendations and regulations

**DOI:** 10.12688/wellcomeopenres.23135.2

**Published:** 2025-03-06

**Authors:** Laura Merson, Karolina Witt, Arishay Hussaini, Ayesha Siddiqui, Eli Harriss, Steve Webb, Patricia Njuguna, Divya K Shah, An-Wen Chan, Robert Terry, Nandi Siegfried, Jeni Stolow, Emmanuelle Denis, Madiha Hashmi

**Affiliations:** 1 Pandemic Sciences Institute, ISARIC, University of Oxford, Oxford, OX3 7LF, UK; 2 Critical Care Medicine, Ziauddin University, Karachi, Sindh, 75000, Pakistan; 3 Bodleian Health Care Libraries, University of Oxford, Oxford, England, OX1 3BG, UK; 4 Australian and New Zealand Intensive Care Research Centre, Monash University, Clayton, Victoria, 3004, Australia; 5 Program for Appropriate Technology in Health (PATH), Nairobi, Kenya; 6 Infectious Disease, Wellcome Trust, London, England, NW1 2BE, UK; 7 University of Toronto, Toronto, Ontario, M5T 3M6, Canada; 8 Special Programme for Research and Training in Tropical Diseases (TDR), Geneva, 1211, Switzerland; 9 South African Medical Research Council, Cape Town, 19070, South Africa; 10 Public Health and Tropical Medicine, Tulane University, New Orleans, Louisiana, 70112, USA

**Keywords:** Quality by design, quality control, research quality, research management, research standards, quality assurance

## Abstract

**Objective:**

Synthesise the published literature and national regulations on infrastructure, capabilities and capacities required to manage and quality assure clinical research.

**Introduction:**

The World Health Assembly (WHA) resolution 75.8 (2022) called “for a strengthened global architecture for coordinated and high-quality clinical trials”. For this remit, infrastructure, capabilities, and capacities needed to design and deliver high-quality clinical trials must be understood and advanced. This rapid scoping review aims to identify the breadth of requirements and recommendations for effective management of clinical trials in regulations, national legislation and the published literature. The findings will be summarised into themes. It will inform a framework for the assessment and development of units undertaking observational studies and interventional clinical trials.

**Inclusion criteria:**

Peer-reviewed literature, grey literature, and national legislation that recommends infrastructure, capabilities, and/or capacities needed to manage and quality assure clinical trials. Publications authored by those who design, manage, fund, sponsor, regulate or oversee clinical trials.

**Methods:**

Peer-reviewed and grey literature will be identified through Medline, Embase, PsycINFO, and Global Health via Ovid; SCOPUS; the Web of Science Core Collection; and the WHO Global Index Medicus using specific field codes to increase the specificity of the search strings. No date, language, or geographic limits will be applied. Deduplicated titles and abstracts will be screened by two blinded reviewers with discrepancies resolved by a third reviewer. Grey literature may be identified through the peer reviewed literature, supplemented with structured searches of Google and DynaMed. National regulations will be sourced online and from available summaries. Full text literature and regulations will be screened by a single reviewer, with proportionate verification by a second reviewer. Data will be extracted and coded for patterns in NVivo software. All items and codes will be summarised using a thematic framework analysis and identify core constructs within each theme.

## Plain language summary

Clinical trials are essential for improving healthcare, but for them to be effective, they need to be high quality. Poor quality trials waste resources and harm public trust in science. During the COVID-19 pandemic, many clinical trials suffered from issues like being underpowered and of low quality. Additionally, clinical trials were underrepresented in low- and middle-income countries (LMICs), with few being led by researchers from those regions.

To address these issues, the World Health Assembly (WHA) and other global bodies have called for stronger global clinical trial infrastructure. The WHA 75.8 resolution, for example, encourages organizations to support high-quality trials, especially in developing countries. Understanding the infrastructure, capabilities, and resources required for running effective trials is essential for improving clinical research globally. This knowledge can help countries and institutions identify gaps and areas for improvement.

Clinical Trials Units (CTUs) are specialized teams or facilities responsible for managing various aspects of clinical trials. These units ensure regulatory compliance, participant recruitment, data management, and trial oversight. CTUs can differ based on the type of clinical trials they handle. For example, different resources are needed for phase I trials, multi-site trials, and trials involving participant recruitment.

There is no globally accepted framework for assessing CTUs. To fill this gap, this review seeks to develop a "CTU Maturity Framework" to evaluate national and institutional resources and provide guidance on strengthening clinical trial operations. A rapid scoping review of literature will inform this framework, allowing for the identification of best practices, regulatory benchmarks, and guidance for clinical trial units worldwide.

This framework aims to improve clinical trials across countries, supporting better health outcomes through high-quality research.

## Introduction

Randomised clinical trials have the potential to provide reliable evidence that can be implemented by health care workers and policy makers to save lives. However, only high-quality clinical trials achieve feasibility, validity, and implementability, and hence capacity for impact. Every trial that falls short in one of these three areas is a wasted opportunity to improve patient outcomes. During the COVID-19 pandemic, the global clinical trials landscape was plagued by underpowered and low-quality studies that wasted limited research resources and threatened public trust in science ^1^. Furthermore, both before and during the COVID-19 pandemic, only a minority of clinical trials were conducted in low- and middle-income countries (LMICs) and even fewer were led by researchers from those countries. This imbalance remains true in the years since the pandemic ^2, 3^.

This has led to calls in the G7 Clinical Trials Charter, the 100 Days Mission, and the World Health Assembly (WHA) to strengthen the global architecture for coordinated and high-quality clinical trials. The WHA 75.8 (2022) resolution invites international nongovernmental organisations and other relevant stakeholders “to support robust, quality clinical trials as well as to strengthen clinical trial research capacities globally, particularly in developing countries…”. Key to delivering on this remit is understanding the infrastructure, capabilities, and capacities needed to effectively plan, operationalise, manage, and/or recruit to clinical trials. The characterisation of these resources will support nations and institutions to identify gaps in their clinical trials ecosystems and define actions to advance priority areas.

In many cases, a specialised team and/or facilities, often called a Clinical Trials Unit (CTU), are dedicated to the planning, coordination, implementation, analysis and/or reporting of clinical trials. A CTU may include personnel with expertise in regulatory and ethics compliance, research methods, statistics, community engagement, participant recruitment and retention, data management or other trial related activities. CTUs that are a part of an institution that sponsors clinical trials may also take responsibility for quality assurance activities. The type of clinical trials planned, and the remit of trial responsibilities define the scope of needs for an individual CTU. For example, different infrastructure, capabilities and capacities will be needed to deliver first-in-human phase 1 clinical trials, to lead and coordinate multi-centre clinical trials, or to enrol participants and collect data as a part of a multi-centre clinical trial. Delineating and describing the range of resources needed for different activities can support targeted strengthening of priority areas.

Many countries have guidance or requirements for the operation of a CTU, for example the Pakistan BioStudy Rules and the India National Accreditation Board for Hospitals & Healthcare Providers Standards for Clinical Trials Sites ^4, 5^. In some countries, CTUs may operate only when externally qualified against a benchmarked standard, for example the United Kingdom Clinical Research Collaboration Registered Clinical Trials Unit Network Key Competencies and Evaluation Framework ^6^. These standards, requirements, and guidance differ between countries and there is no globally accepted framework. This rapid scoping review aims to inform the development of a maturity framework that supports the assessment of national and institutional clinical research resources and plan for improvements to deliver robust, quality clinical trials and strengthen the broader clinical research ecosystem. 

As a baseline for development of these metrics, this rapid scoping review will identify the spectrum of requirements and recommendations for effective management and implementation of high-quality clinical trials across national authorities and in the published literature. The aim is to capture the breadth of unique and overlapping resources, then summarise their contents by theme. We have selected a rapid scoping review method due to the breadth of the research question, our intent to map the range and gaps in topics, and our focus on qualitative synthesis. A preliminary search of MEDLINE, PROSPERO, the Cochrane Database of Systematic Reviews and JBI Evidence Synthesis was conducted and no existing or registered systematic reviews or scoping reviews on the topic were identified. Our review will identify national regulations and other publications that describe benchmarking, capacity, quality, capabilities, or infrastructure, for clinical trials to inform global clinical trial practice. Publications authored by individuals from all types of institutions that design, manage, fund, sponsor, regulate or otherwise oversee clinical trials will be the focus. An initial review of PubMed and Google have identified a wide variety of material on these topics, ranging from recommendations drawn from a single clinical trial to national accreditation programmes.

By consolidating the experience, guidance, and interests of the global clinical trials community, we will promote an inclusive starting point for the development of maturity metrics for clinical trials units. The methods employed to control and assure clinical trial quality have many similarities with those used in other forms of clinical research, such as observational studies. Consequently, we anticipate that the findings of this review will identify key maturity metrics for research units engaged in a range of clinical studies which may encompass, but are not limited to, clinical trials. The findings of this review will assist national authorities and research institutions in understanding the critical role of quality in clinical research and enhance their capacity to achieve it. Literature from many disease areas, geographic regions, regulatory contexts, and trial phases will bring a diversity of perspectives to inform the range of capacities, capabilities and infrastructure needed to plan and execute high quality research, with a focus on clinical trials.

## Review questions

What is the breadth of requirements, regulations, recommendations and good practice guidance published on optimising the quality of clinical trial management? What infrastructure, capabilities and capacities do those who manage, oversee, and fund clinical trials associate with the maturity of the units that support their delivery?

## Eligibility criteria

### Participants

Publications regarding the management of clinical trials in any population, authored by individuals, institutions or organisations who participate in the design, delivery, management, analysis, oversight, funding, sponsorship, or regulation of trials.

### Concept

Peer-reviewed literature, grey literature, and regulations that describe or recommend infrastructure, capabilities, and/or capacities needed to manage and quality assure clinical trials.

### Context

No date, geographic or language limitations will be applied to the search criteria. Publications originating from clinical trials regulations or experience will be included from a diversity of contexts, including low- and middle-income countries. All types of authoring institutions will be eligible, including academic, industry, government, non-government or others without exclusion.

### Sources

This rapid scoping review will consider a broad range of literature, including qualitative or quantitative research, opinion papers, case reports, first-hand accounts, and guidance or recommendations issued by health or research authorities, professional societies, research units or networks, government, or other authorities. Primary or secondary reports will be acceptable. News items such as journalistic articles, press releases, and media reports that do not provide primary research, formal guidance, or authoritative recommendations from regulatory bodies, research institutions, or professional organizations will not be included.

## Methods

This protocol is based on the Joanna Briggs Institute (JBI) scoping review protocol template and adheres to the Preferred Reporting Items for Systematic Reviews and Meta-analyses extension for scoping review (PRISMA-ScR) guidance ^7, 8^. A review protocol developed and published on Open Science Framework in February 2024 can be accessed at https://osf.io/p6kyd/ ^9^.

### Search strategy

An initial scoping of PubMed and Google will be undertaken to identify articles on the topic. The text words contained in the titles and abstracts of relevant articles, and the index terms used to describe the articles will be used to develop a full search strategy. An information specialist will search the following databases from inception to the search date for relevant references: Medline, Embase, PsycINFO, and Global Health all via Ovid; SCOPUS; the Web of Science Core Collection; and the WHO Global Index Medicus. No limits will be applied to the search results. The databases will be searched using specific field codes to increase the specificity of the search strings to make the overall total number of results to screen manageable given the resources available. The Ovid databases will be searched using the title and abstract fields for references related to clinical research and trials, including terms such as study, studies, and trial; quality and standards, including benchmarking, standards, accreditation, and framework; infrastructure and capacity, including unit, hub, capacity, systems, and metrics; and governance and management, including governance, integrity, management, quality, capabilities, guidance, and skills. Proximity commands will be applied to refine the search and improve specificity.

SCOPUS, the Web of Science Core Collection, and the WHO Global Index Medicus will be searched in the title field only for the same reason, using proximity commands where available. The TRIP Database will be searched specifically for Regulatory Guidance. This prioritising search method and description meets the recommendations by Speckemeier *et al.* ^10^. The detailed strategies are available in the extended data.

Websites and reports, including WHO reports, the Office for Human Research Protections International Compilation of Human Research Standards, and the National Institute of Health’s ClinRegs Database, will be reviewed to identify legislative documents and regulatory frameworks. A combination of structured keyword searches and manual browsing will be used to ensure comprehensive coverage. National regulations will be identified via the websites of the relevant authorities. For screening, the same eligibility criteria will be applied for inclusion of recommendations as above.

Following an initial screening of publications, a grey literature search will be conducted from the sources above, Google and DynaMed searches will be designed to identify additional, unique literature. The search will be conducted across i) internet search engine (Google advanced search) and ii) associated references cited in identified documents. The eligibility criteria undertaken for grey literature will be the same as above. Search terms will include, “clinical trials units” combined with terms relevant to the inclusion criteria, including “regulations”, “recommendations”, "gold standard”, and “maturity framework”. Search will be limited to the first 5 pages of Google search results and the date recorded for transparency.

### Screening and selection of eligible publications

All peer-reviewed references will be exported to EndNote 21 (Thomson Reuters, New York, NY), and deduplicated in Rayyan ( https://www.rayyan.ai). Pilot screening will be done by all team members to develop a sample list of topics and content for inclusion or exclusion (see extended data). When a draft list of eligible content by topic is agreed across the team screening will begin with a fresh list of deduplicated references.

Titles and abstracts will be screened by two independent reviewers against the eligibility criteria in a single cycle. Each reviewer’s decision will be blinded from other reviewers. The list of eligible content by topic will be consulted and collectively iterated according to the eligibility criteria as screening progresses. Discrepancies in the selections of two reviewers will be scrutinised by a third individual to make a final decision, in consultation with the two reviewers. Where suitability is unclear, the team will include the publication in the list for full-text review.

Challenges to exclusion decisions will be discussed between reviewers for a final decision and strengthening consensus across the team. Reasons for exclusion will be recorded and reported alongside all details of identified, screened, included and excluded publications in a Preferred Reporting Items for Systematic Reviews and Meta-analyses extension for scoping review ( PRISMA-ScR statement) flow diagram ^8^. As a quality assurance measure, ten percent of excluded publications will be reviewed by a second reviewer. 

Full-text publications or website links (URLs) will be retrieved for all selections approved by two or three reviewers during the title and abstract review process. If retrieval of the full text is not possible, the abstract will be assessed for data extraction. 

### Data extraction

A codebook of topics and categories will be developed based on the TDR Research Competencies Framework ^11^. The codebook will additionally include other codes generated by the research team during title and abstract screening. Individual researchers will code relevant text using cloud-based qualitative data analysis software ( NVivo14 software ( Lumivero) version 14.23.3).

Each full-text publication will be reviewed for inclusion and data extraction by a single reviewer from one of three institutions: OUCRU (Vietnam), University of Oxford (UK), or Ziauddin University (Pakistan). Papers in languages other than English, will be translated into English and the translated version will be uploaded into the NVivo cloud. Translations will be conducted using a combination of automated tools and, where feasible, manual verification. Specific focus will be given to three topics of special interest to the WHO team involved in this effort: (A) phase I studies, (B) international and multi-site trials, and (C) patient recruitment and management in clinical trials.

The codebook will be used to generate a preliminary results framework. This framework will be updated iteratively, and collaboratively as new topics and keywords are identified. Coding will be reviewed a second time by a second researcher or "Code Specialist" to ensure consistency and rigour across the analysis. Any coding discrepancies between the reviewers will be resolved through discussion. At least 10% of papers will be evaluated jointly during team meetings to ensure intercoder reliability. The process of full text paper review will be coordinated, and all activity will be monitored using a shared project tracker, which will be regularly updated with progress and feedback to the Project Advisory Committee on a weekly basis.

### Data analysis and presentation

Data will be synthesised into themes by comparing, connecting, and combining keywords and category codes. Data will be organised thematically and summarised inclusively to present all unique recommendations within each theme. Results will be structured into a framework of infrastructures, capacities and capabilities for each identified theme. Data gaps within the framework will be identified. Data will be descriptively analysed by these categories where appropriate. A qualitative narrative summary will accompany the tabulated results and will describe how the results relate to the development of a maturity framework for units managing clinical trials and other types of clinical research. A final report of the structured results will be presented to working groups contributing to a maturity framework for clinical trial units.

No quality assessment of the document contents will be conducted as it is deemed unsuitable for the inclusive methodology applied and no comprehensive system for critically appraising this range of literature was identified.

## Ethics and consent

Ethical approval and consent were not required.

## Data Availability

No data are associated with this article. OSF: Infrastructure, capabilities, and capacities required for clinical trials design and delivery: a rapid scoping review of recommendations and regulations. https://doi.org/10.17605/OSF.IO/P6KYD ^9^ This project contains the following extended data: 1. Detailed search strategy 2. The topics and category framework for data coding OSF: PRISMA-ScR checklist for ‘Infrastructure, capabilities, and capacities required for clinical trials design and delivery: A rapid scoping review of recommendations and regulations’ https://doi.org/10.17605/OSF.IO/P6KYD ^9^ Data are available under the terms of the Creative Commons Attribution 4.0 International license (CC-BY 4.0).
