# Supplementary material for: Infrastructure, capabilities, and capacities required for clinical trials design and delivery: A rapid scoping review of recommendations and regulations
Source: Wellcome Open Res. 2025 Mar 6;9:729. Originally published 2024 Dec 19. [Version 2] doi: 10.12688/wellcomeopenres.23135.2 (PMC11923532; doi:10.12688/wellcomeopenres.23135.2)
Supplement: Peer review history

# Reviewer report: Reviewer response for version 2

**DOI:** 10.21956/wellcomeopenres.26355.r120247

**Published:** 2025-03-19

**Authors:** William J Cragg

**Affiliations:** 1 University of Leeds, Leeds, England, UK

I have reviewed the responses and have nothing else to raise *e*xcept in relation to my second point – the text ‘trials involving participant recruitment’ is actually in the plain language summary rather than the abstract. I think that comment would still be worth the authors’ attention, but otherwise I have nothing to raise.

Is the study design appropriate for the research question?

No

Is the rationale for, and objectives of, the study clearly described?

Yes

Are sufficient details of the methods provided to allow replication by others?

Partly

Are the datasets clearly presented in a useable and accessible format?

Not applicable

Reviewer Expertise:

NA

I confirm that I have read this submission and believe that I have an appropriate level of expertise to confirm that it is of an acceptable scientific standard.

# Reviewer report: Reviewer response for version 1

**DOI:** 10.21956/wellcomeopenres.25475.r116090

**Published:** 2025-01-22

**Authors:** William J Cragg

**Affiliations:** 1 University of Leeds, Leeds, England, UK

Thanks for the invitation to review this paper about an important project. It is clear overall, and I just have a few comments for your consideration below.

1. General comment: I think the theme of risk proportionality could come through more strongly in this work (as per principle 7 of the new ICH GCP revision). I think a negative outcome of this work would be recommendations that say or imply that clinical trials can only be run using a large amount of resource. Risk-proportionality is also key to delivery as it enables us (humanity) to do more trials, more quickly and more cheaply (I appreciate quality is the other important aspect to cover there). In other words, you might give as much emphasis to 'efficient' trial management as you do to 'effective' trial management. I don't mind what you do in response to this comment.
2. Abstract: 'trials involving participant recruitment' - this sounds like all trials; could you explain what you mean here?
3. Introduction: 'this imbalance remains true in the years since the pandemic' - this claim needs a reference.
4. Eligibility criteria: I wonder if you might miss relevant information by focusing only on published information. You mention the UKCRC Registered CTU Network, and their registration criteria do appear to be public. However, might there be other, similar networks in other countries whose criteria are not public, but which might be obtainable if you ask?
5. Eligibility criteria: 'authored by individuals, institutions or organisations who participate in the design, delivery, management, analysis, oversight, funding, sponsorship, or regulation of trials' - how will you verify this criterion?
6. Eligibility criteria - sources: 'news items will not be included'. How are you defining 'news items'?
7. Methods, search strategy: I think your decision to have no date limits on search results need justification. E.g. would a result from 30+ years ago still be useful?
8. Methods, search strategy: scoping review methodology tends to include a consultation with relevant experts to find additional information/results. If you are not including this, you should justify why not.
9. Methods, search strategy: will you include all countries in your search for relevant legislation?
10. Methods, search strategy: sentence starting 'the title and abstract fields' - this sentence is too long and unnecessarily writes out lots of the search strategy. Please consider another way to present this information.
11. Methods, search strategy: 'websites and reports…will be reviewed to identify legislative documents and regulatory frameworks'. 'Will be reviewed' is a bit vague, here; will there be a search method? Or is it simple 'hand searching' in this case?
12. Methods, search strategy: recording the date of the Google search is good, but the location of the search may affect the results, too. Might you record this as well?
13. Methods, data extraction: might a reference/link be needed for 'TDR Research Competencies Framework'?
14. Methods, data extraction: 'papers in languages other than English will be translated…' - how?
15. Methods, data analysis: what will you do if sources disagree on a particular point?
16. Methods, data analysis: 'no quality assessment…was conducted' - why past tense?

Is the study design appropriate for the research question?

No

Is the rationale for, and objectives of, the study clearly described?

Yes

Are sufficient details of the methods provided to allow replication by others?

Partly

Are the datasets clearly presented in a useable and accessible format?

Not applicable

Reviewer Expertise:

NA

I confirm that I have read this submission and believe that I have an appropriate level of expertise to confirm that it is of an acceptable scientific standard, however I have significant reservations, as outlined above.

# Response

**Published:** 2025-02-27

**Authors:** Ayesha Siddiqui

**Affiliations:** Critical Care Medicine Research, Ziauddin University, Karachi, Sindh, Pakistan

1. Thank you for this insightful comment. We agree. Though this is not a feature of the protocol, we will be sure to embed this reflection into our reporting of findings and of the Maturity Framework in general.

 2. Thank you for your comment. We have reviewed the abstract but could not find this exact sentence. Could you kindly clarify which part you are referring to?

 3. Thank you, this has been added. 

 4. Our review includes grey literature searches, which allow us to capture non-peer-reviewed sources such as institutional reports, regulatory documents, and other publicly accessible materials. While unpublished criteria will be available by direct inquiry, we acknowledge that such data would be difficult to standardize or validate across multiple contexts. So, our approach prioritizes publicly available sources while recognizing that certain non-public information may remain outside the scope of this review.

 5. As a scoping review, our focus is on mapping existing literature rather than independently verifying authors’ roles. We will rely on the information provided within publications, considering an article eligible if the authors’ affiliations and content indicate involvement in clinical trial design, management, oversight, funding, sponsorship, or regulation.

 6. Thank you, this has been defined as news items such as journalistic articles, press releases, and media reports that do not provide primary research, formal guidance, or authoritative recommendations from regulatory bodies, research institutions, or professional organizations.  

*7. *Yes, our approach is that older documents may provide historical context on evolving regulations and practices, as well as be relevant in some resource limited settings.

 8. Thank you, as this review prioritizes systematically published guidance and regulations. For developing the maturity framework, subsequently, expert input will be sought.

 9. Yes, all countries will be included in the search. 

 10. Thank you, we agree, this has been changed to: The Ovid databases will be searched using the title and abstract fields for references related to clinical research and trials, including terms such as study, studies, and trial; quality and standards, including benchmarking, standards, accreditation, and framework; infrastructure and capacity, including unit, hub, capacity, systems, and metrics; and governance and management, including governance, integrity, management, quality, capabilities, guidance, and skills. Proximity commands will be applied to refine the search and improve specificity.

 11. This sentence has been added to eliminate ambiguity: A combination of structured keyword searches and manual browsing will be used to ensure comprehensive coverage.

 12. Yes, we will record date, time and location. 

 13. Thank you, this has been added. 

 14. We have added: Translations will be conducted using a combination of automated tools and, where feasible, manual verification.

 15. Thank you for your comment. As a scoping review, our focus is on mapping existing perspectives rather than resolving discrepancies. We will ensure that differing viewpoints are documented objectively and presented transparently in our findings.

 16. Thank you, this has been corrected.

# Reviewer report: Reviewer response for version 1

**DOI:** 10.21956/wellcomeopenres.25475.r116965

**Published:** 2025-01-20

**Authors:** James Spicer

**Affiliations:** 1 King's College London, London, UK

The authors propose to synthesise the published literature and national regulations on infrastructure, capabilities and capacities required for clinical research. The scope of the literature and national regulations to be searched is broadly defined and appropriate. It will be important to review these critically, particularly in the case of the regulatory framework. A survey of the regulatory requirements governing trials will be of value, especially where there is divergence even in otherwise similar healthcare economies, but these should be evaluated, not simply catalogued. Regulatory requirements in many jurisdictions are too regimented in some scenarios, and may stifle innovation and slow development of advances in care [Rule S et al. (2019)]

 Key aspects for focus should include examination of the role and practicalities of allowing for proportional tolerance of risk, in particular differentiating the widely differing contexts for research involving healthy volunteers and that recruiting patients with immediately life-threatening diagnoses such as advanced malignancy. In various jurisdictions this has been attempted [“Risk-adapted approach to clinical trials and risk assessments”, MHRA 2022; ‘Risk proportionate approaches in clinical trials”, EU 2017], but many argue that progress in this area has to date been too conservative.

 Other issues for critical review include excessively narrow trial eligibility, driven by a simplistic aspiration for homogeneity in study population, but instead jeopardising the real-world validity of results [AACR Cancer Disparities Progress Report 2024].

 The authors allude to the pressures of the COVID-19 pandemic on trial design and conduct, and it will be interesting to see whether this scoping review supports the suggestion that these arose from a deficiency in trial infrastructure, or whether in part they were a result of unrealistic expectations and §over-allocation of resource, to the detriment of deferred and discontinued non-pandemic-related clinical research.

Is the study design appropriate for the research question?

Yes

Is the rationale for, and objectives of, the study clearly described?

Yes

Are sufficient details of the methods provided to allow replication by others?

Yes

Are the datasets clearly presented in a useable and accessible format?

Yes

Reviewer Expertise:

Clinical trial design, delivery and governance.

I confirm that I have read this submission and believe that I have an appropriate level of expertise to confirm that it is of an acceptable scientific standard.

## References

[1] : Bureaucracy is strangling clinical research. *BMJ* .2019;364: 10.1136/bmj.l1097 l1097 10.1136/bmj.l1097 30867145

# Response

**Published:** 2025-02-27

**Authors:** Ayesha Siddiqui

**Affiliations:** Critical Care Medicine Research, Ziauddin University, Karachi, Sindh, Pakistan

Thank you for your detailed and thoughtful feedback. We appreciate the emphasis on critically reviewing regulatory frameworks rather than simply cataloguing them. While our primary aim is to map existing regulations and recommendations, we recognize the importance of highlighting areas where rigid regulatory structures may hinder trial innovation and efficiency. We will ensure that our synthesis acknowledges divergences in regulatory approaches, including those that allow for risk-proportional tolerance, particularly in different research contexts such as healthy volunteer studies versus trials involving critically ill patients. To your point on eligibility criteria and the potential limitations of overly narrow selection criteria, where relevant, we will capture discussions in the literature regarding the balance between scientific rigor and real-world applicability in clinical trials. Regarding the impact of the COVID-19 pandemic on trial infrastructure and conduct, we agree that assessing whether challenges stemmed from systemic deficiencies or shifting research priorities is important. However, as our scoping review is intended to inform a maturity framework, it does not fall within our remit to evaluate the underlying causes of these changes. We will document reported changes in trial conduct and infrastructural requirements in crisis and pandemic situations. Thank you again for your constructive comments, which will help refine the focus of our review.
